# Quality Parameters of Wheat Bread with the Addition of Untreated Cheese Whey

**DOI:** 10.3390/molecules26247518

**Published:** 2021-12-11

**Authors:** Charikleia Tsanasidou, Ioanna Kosma, Anastasia Badeka, Michael Kontominas

**Affiliations:** Laboratory of Food Chemistry and Technology, Department of Chemistry, University of Ioannina, 45110 Ioannina, Greece; xaroulatsan@gmail.com (C.T.); i.kosma@uoi.gr (I.K.)

**Keywords:** wheat bread, untreated cheese whey, bread quality parameters, shelf-life of bread

## Abstract

Τhe present study was carried out to evaluate wheat bread of three different flour compositions prepared by replacing water with untreated cheese whey (WCB). Bread prepared with water was taken as the control (CB). All breads were stored at 24 ± 1 °C for up to 6 days. Microbiological, physicochemical, and sensory analyses were determined as a function of storage time. WCB had lower total viable counts (TVC) (3.81 log cfu/g for CB and 2.78 log cfu/g for WCB on day 2 of storage) and showed delayed mold growth by 1 day (day 4 for CB and day 5 for WCB). WCB also had lower pH (5.91 for CB and 5.71 for WCB on day 0), higher titratable acidity values (TTA) (2.5–5.2 mL NaOH/10 g for CB and 4.5–6.8 mL NaOH/ 10 g for WCB), and higher protein content (PC) (PC 7.68% for CB and 8.88% for WCB). WCB was characterized by a more intense flavor, reduced hardness but similar cohesiveness, springiness, and adhesiveness compared to CB. Based primarily on sensory (appearance/mold formation) data, the shelf life of WCB was 4–5 days compared to 3–4 days for CB stored at 24 ± 1 °C. The proposed use of whey in bread preparation contributes decisively to the environmentally friendly management of whey.

## 1. Introduction

Whey is defined as the milk serum remaining after separation of the cheese curd, resulting from the coagulation of milk proteins through the use of either acid or proteolytic enzymes [1]. Whey, a by-product of cheese making, is distinguished into sweet whey (from the precipitation of casein by rennet) and acid whey (coagulation of proteins through the addition of acid) [2]. Whey is composed of lactose (70–72% of the total solids), whey proteins (8–10%), and minerals (12–15%) on dry basis [1].

Due to its high biological oxygen demand (BOD) and chemical oxygen demand (COD) content, whey causes an enormous amount of environmental pollution [2]. However, it comprises an excellent source of functional proteins and peptides, lipids, vitamins, minerals, and lactose [3]. Consequently, discarding whey constitutes a significant loss of potential nutrients and energy [1]. Moreover, compelling evidence in the literature supports the exceptional nutritional quality of whey protein compared to common dietary protein sources. The Biological Value of whey proteins exceeds that of egg protein (reference protein) by ca. 5%, due to its content of essential amino acids and specifically of the branched chain amino acids (leucine, isoleucine, and valine) [3].

Whey is widely used in the production of whey cheeses, many of which have distinct names based on the region or country of origin such as Myzithra, Manouri, Ricotta, etc. It is also used to commercially produce whey protein bars and beverages used as dietary supplements by athletes, resulting in maximum physical performance [4]. According to USADEC [4], concentrated whey can be used for texture enhancement in meat and poultry products, color enhancement in bakery and confectionery products substituting the action of milk or eggs, etc.

Bread constitutes one of the most important staple foods, being a good source of macronutrients (carbohydrates, proteins) and micronutrients (vitamins, minerals). However, it is susceptible to mold growth and has a short shelf life (typically of 3–7 days) when stored at room temperature due to its high moisture content (about 40%) and water activity (about 0.94–0.97) [5]. For this reason, several additives (mainly sorbate and propionate salts) are added in order to increase bread shelf life and prevent its microbiological spoilage [6].

The time-dependent loss of bread quality (deterioration of texture, taste, and aroma) is described as “staling”. This phenomenon has been mostly attributed to the retrogradation of starch, but it is also related to moisture loss. Both starch retrogradation and moisture loss equally contribute to bread staling and result in the hardening of bread texture [7].

Flavor, on the other hand, comprises an equally significant sensory parameter for the acceptability of bread by consumers. A large number of volatile compounds are responsible for the aroma of fresh bread including alcohols, aldehydes, esters, pyrazines, pyrrolines, etc. [8].

To the best of our knowledge there are no data available in the literature involving the direct use of untreated cheese whey for the production of leavened wheat bread. Therefore, the objective of the present study was to evaluate, on a qualitative and quantitative basis, commercial wheat breads prepared by replacing water with untreated cheese whey. At the same time, the proposed application comprises a novel use of cheese whey contributing to the solution of the whey disposal problem.

## 2. Results and Discussion

### 2.1. Determination of Microbiological Parameters

**Figure 1:**
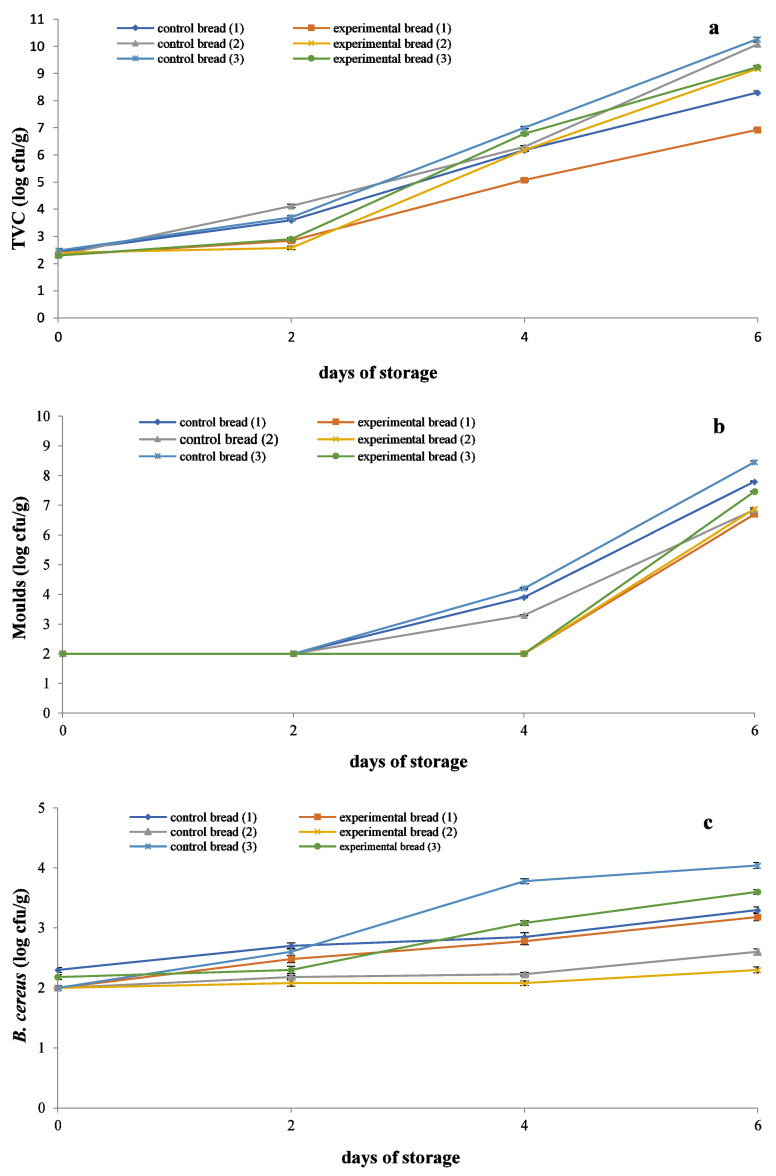
Effect of water substitution with untreated cheese whey and storage time on (**a**) TVC, (**b**) molds, and (**c**) *Bacillus cereus* of sliced bread stored at ambient temperature. Breads: (1) F70/WWF, (2) F70/FM, and (3) F70, stored at ambient temperature.

TVC, yeasts/molds, and *Bacillus cereus* counts for all breads as a function of storage time are shown in Figure 1a–c.

The initial TVC value was 2.40 log cfu/g for control breads and 2.30 log cfu/g (*p* > 0.05) for experimental breads. TVC exceeded the value of 6.00 log cfu/g, considered as the upper microbiological limit for bakery products, as defined by the ICMSF [9] on day 3–4 for all three control breads and two of the three experimental breads (F70/FM and F70). Experimental bread F70/WWF reached 6.00 log cfu/g on day 5 of storage. Likewise, Karaoglou et al. [10] reported a TVC of ca.7 log cfu/g after 5 days of storage at 20 °C for white pan bread. After day 3, there was a statistically significant difference in TVC (*p* < 0.05) among the three experimental breads with the exception of breads 2: F70/FM and 3: F70, which showed no differences in TVC on day 6 of storage. Latou et al. [11] reported a TVC of 7 log cfu/g in control bread packaged in high-barrier PET-SiOx//LDPE pouches, which were stored at 20 °C on day 7 of storage. Differences in time to reach 7 log cfu/g in both the above studies may be related to the lower storage temperature used compared to that in the present study (20 vs. 24 °C) as well as the high barrier packaging used in the latter study providing better protection from mold growth, which is the main limiting factor in bread shelf life.

Yeasts and molds were under the method detection limit (2 log cfu/g) [9] until day 2 for the control breads and day 4 for the three experimental breads. On day 4 of storage, yeasts and molds counts increased to 3.30–4.20 log cfu/g for control breads. On day 6 of storage, yeasts and molds counts reached 6.80–8.40 log cfu/g for control breads and 6.70–7.40 log cfu/g for experimental breads. Sensory evaluation (see below) showed that mold growth became visible when yeast and mold counts were ≥4.00 log cfu/g ca. on day 4 for control breads and ca. on day 5 for experimental breads. Statistically significant differences in yeasts and mold counts (*p* < 0.05) were recorded after day 5 for breads 1 and 2 vs. bread 3, while no differences were recorded between breads 1 and 2 during the same period. Similar to our results, Latou et al. [11] observed visible mold growth in control bread when the mold population was ca. 4.00 log cfu/g on day 5 of storage at 20 °C. The yeasts and molds population reported on day 5 is in agreement with the value reported in the present study given the difference in storage temperature of bread between the two studies (24 vs. 20 °C). Divya and Rao [12] studied the effect of adding whey concentrate (15% total solids) from Indian paneer (cottage cheese) in the bread flour. The reported mold counts for the prepared bread ranged from 1.65 log cfu/g (day 0) to 2.34 log cfu/g (day 4), and for the control bread ranged from 2.18 log cfu/g (day 0) to 2.81 log cfu/g (day 4). Data of the above study are in good agreement with that of the present study considering the differences in bread storage temperature. Mold growth in commercial wheat bread is a common phenomenon, depending on product storage temperature and relative humidity, as early as 3–4 days and in home-baked bread even sooner where preservatives are not used.

According to the EFSA [13], the threshold level of *B. cereus* counts associated with foodborne diseases is 5.00 log cfu/g. *B. cereus* counts increased from <2.00 log to 3.31 log cfu/g for control breads and from <2.00 to 3.03 log cfu/g for experimental breads during 6 days of storage. Statistically significant differences in *B. cereus* counts (*p* < 0.05) were recorded among the three experimental breads after day 2 of storage. Latou et al. [11] studied the contamination of control bread by *B. cereus* during storage and concluded that its counts increased from <2.00 log cfu/g (day 0) to 5.60 log cfu/g (day 9 of storage). A similar increasing tendency was recorded in the present study for control breads during the 6-day storage period.

Microbiological analyses showed that incorporating whey to bread reduced TVC, mold, and *B. cereus* count, delaying mold growth by 1 day (day 4 for control breads and day 5 for experimental breads). Such reductions may be attributed to the lower pH and higher TTA of experimental breads compared to control breads caused by the addition of whey (see below). In addition, it is possible that the antimicrobial activity of whey proteins such as lactoferrin, lactoperoxidase, and immunoglobulins may be involved in microbial growth reduction. According to Smithers [3], the proteolytic digestion of lactoferrin (by pepsin and chymosin) produces lactoferricin, which is a peptide with potent antimicrobial and antifungal activity.

### 2.2. Determination of Physicochemical Changes

#### 2.2.1. pH and TTA

The pH of the untreated cheese whey was recorded to be 6.00 ± 0.01 pH measurements showed a statistically significant difference (*p* < 0.05) between control breads and whey-enriched breads. The mean pH value was 5.91 for control breads and 5.71 for whey-enriched breads on day 0. Differences in pH between control and experimental breads are obviously due to the lower pH of the added whey.

**Figure 2:**
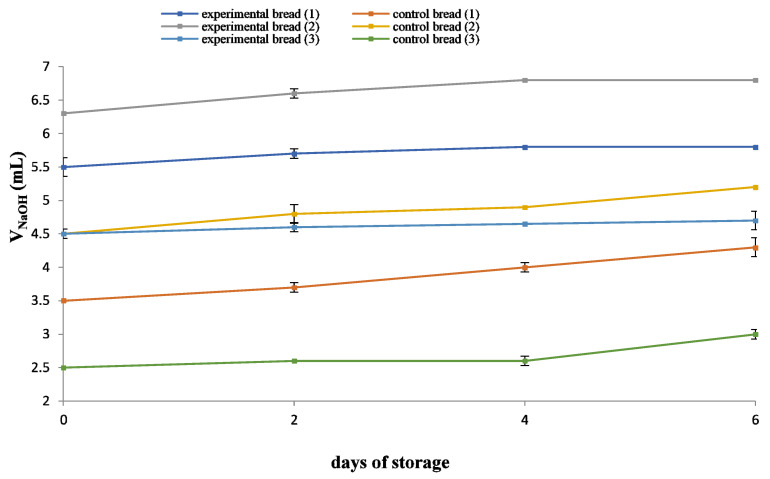
Effect of water substitution with untreated cheese whey and storage time on total titratable acidity (TTA) of sliced bread stored at ambient temperature. Breads: (1) F70/WWF, (2) F70/FM, (3) F70, stored at 24 ± 1 °C.

The TTA of the untreated cheese whey was calculated to be 0.12% *w*/*v* in lactic acid. The results of TTA (mL NaOH 0.1 N) determination in all breads are shown in Figure 2. The addition of whey increased the TTA of breads, remaining stable over the storage time. The TTA range was 2.5–5.2 mL NaOH/10 g of bread for control breads and 4.5–6.8 mL NaOH/10 g of bread for whey-enriched breads. Changes in TTA between the experimental and control breads of a given composition were statistically significant (*p* < 0.05) with experimental breads exhibiting higher acidities. Such differences are owed to the acidic nature of whey in experimental breads. The order of increasing experimental bread TTA based on flour composition was F70 < F70/WWF < F70/FM. The results of pH and TTA values are in agreement with the microbiological data, since the increased acidity of experimental breads is associated with reduced microbial growth. Statistically significant differences in TTA (*p* < 0.05) were recorded among the three experimental breads throughout storage.

#### 2.2.2. Determination of Protein and Lactose Content

The protein content of the untreated whey was 1.31%. The protein content range was 6.78–8.16% for control breads and 8.69–9.08% for whey-enriched breads. The lactose content of untreated whey was 5.43%. Lactose content was 0.35% for F70/WWF, 0.71% for F70/MF, and 0.35% for F70 bread.

The increase in protein content is in accordance with the results reported by Constandache [14] by substituting water with whey protein concentrate in breadmaking. El-Batawy et al. [15] concluded that substituting water with cheese whey at 50% and 100% in the preparation of Egyptian Baladi flat bread increased its protein content from 7.32% to 7.93% and 8.22%, respectively. Divya and Rao [12] reported that the protein content of bread increased from 9.45% (control bread) to 9.96%, incorporating whey concentrate (15% total solids) from Indian paneer (cottage cheese) in the flour.

#### 2.2.3. Semi-Quantitative Determination of Volatile Compounds

The most abundant volatile compounds identified (Table 1) in freshly baked (day 0) bread, (both experimental and control) were: ethanol, 2-methyl-1-propanol, 3-methyl-1-butanol, 2-methyl-1-butanol, 2,3-butanediol, 3-methyl-1-butanal, hexanal, heptanal, nonanal, acetoin, methyl butanoate, hexane, and limonene. Minor differences were observed in the volatiles’ profile of all three breads. On day 4 of storage, a drastic decreasing trend was observed (for both experimental and control breads) for most volatile compounds with a few exceptions such as that for nonanal, which increased during bread storage and those of hexane, limonene, 2,3-butadienol, and methyl butanoate that remained more or less the same.

Aroma compounds of bread originate mostly from fermentation (40%), Maillard reactions (33%), and the oxidation of lipids (27%) [16]. The identified compounds produced from the glycolysis of pyruvic acid during fermentation [16] were ethanol, 2,3-butanediol, 1-hexanol, benzaldehyde, and acetoin, while 2-methyl-1-propanol, 3-methyl-1-butanol, 2-methyl-1-butanol, phenylethanol, 2-methyl-1-propanal, 3-methyl-1-butanal, 2-methyl-1-butanal, and phenylacetaldehyde were derived from a secondary fermentation reaction, namely the Ehrlich pathway [16]. The last four compounds may also originate from the Strecker degradation of the Maillard reaction [17]. Hexanal, heptanal, octanal, and nonanal were products of lipid oxidation [16].

The oxidation of aldehydes to acids subsequently led to the formation of the following esters identified: methyl butanoate, ethyl butanoate, methyl hexanoate, and ethyl octanoate [18]. Moreover, these esters can derive from a reaction in the yeast cell (catalyzed by acetyl-transferases) between acetyl co-enzyme, derivatives of fatty acids (C6–C10), and alcohols (mainly ethanol) [19]. The hydrocarbons identified in all breads are hexane, heptane, nonane, decane and 2-pentyl-furan; of these, 2-pentyl-furan, and heptanes are products of lipid oxidation (mainly of linoleic acid) [20].

The identified terpenes included a-pinene, limonene, farnasene, ocimene, and p-cymene. The relatively high number and content of terpenes are probably due to the presence of olive oil used for breadmaking. In the present study, pyrazines were not identified, except for 2-ethyl-5-methyl-pyrazine and 2,5-dimethyl-pyrazine in the control bread (F70) on day 0. A possible explanation is that after baking, pyrazines disappear very rapidly via evaporation from the bread sample.

The identified compounds associated to bread aroma [16] were 3-methyl-1-butanol, 2-methyl-1-butanol (malty odor), phenylethanol (flowery aroma), 2-methyl-1-propanal (malty odor), 3-methyl-1-butanal (malty odor), phenyl-acetaldehyde (honey-like odor), octanal (citrus odor), and acetoin (butter, cream odor). All the above compounds, except for 3-methyl-1-butanol, 2-methyl-1-butanol, and phenylethanol, were found at higher concentration in the experimental bread compared to the control during storage.

Volatile compound analysis showed that experimental breads were characterized by a more intense aroma compared to control breads (9065.7 vs. 8604.1 μg/kg on day 0 and 4596.9 vs. 3943.6 μg/kg on day 4 of storage). A higher loss of numerous volatile compounds as well as an increase in the concentration of off-flavor compounds (produced from lipid oxidation during storage) was observed in control vs. experimental breads. The observed loss of aldehydes, produced by the Strecker degradation (2-methyl-1-propanal, 3-methyl-1-butanal and 2-methyl-1-butanal), was also reported by Plessas et al. [21] after bread storage for 5 days.

In agreement with our results, Quilez et al. [22] reported that a high content of alcohols, esters, and ketones positively correlated with the desirable aroma/flavor of prebaked commercial type-baguette bread during storage.

The results of the present study confirm those of Rychlik and Grosch [23] regarding the formation of aromatic compounds (contributing mainly to crust flavor) such as 2-methyl-1-propanal, 3-methyl-1-butanal, and 2-methyl-1-butanal. Of the aldehydes reported by Pico et al. [16] in wheat bread flavor profile (heptanal, octanal, 2-octenal, nonanal, 2-(*E*)-nonenal, and 2,4-(*E*,*E*)-decadienal), only heptanal, octanal, and nonanal were identified in the present study. The reported volatile compounds in the present study are also in agreement with those of Latou et al. [11] for wheat bread, except for 2-furancarboxaldehyde, ethyl acetate, acetic acid, and pentanoic acid.

#### 2.2.4. Color Measurement

Color measurements showed that whey-enriched breads were, in most cases, characterized by a brighter, more yellowish crumb and a darker, less yellowish crust compared to control breads (*p* < 0.05) (Table 2). The yellowish hue of the breadcrumb can be attributed to vitamin B2 (riboflavin) in milk, which is transferred to cheese whey by ca. 70–80% [24]. The darkness of the crust is associated with the presence of lactose, which acts as a browning agent through Maillard reactions and caramelization [4]. The observed lighter bread crumb color compared to that of the bread crust due to the direct exposure of the bread surface to heat during baking is in agreement with the findings of Kadharmestan et al. [25]. Finally, the yellow hue of bread observed in the present study was also reported by Divya and Rao [12], who incorporated whey concentrate (26% total solids) from Indian paneer (cottage cheese) in the flour used for the preparation of bread (only sensory data).

#### 2.2.5. Measurement of Mechanical Properties

Texture analysis showed that for a given sampling day, hardness was reduced (*p* < 0.05) in two of the three types of experimental breads (F70/WWF and F70) compared to control breads (Table 3). This phenomenon is justified probably by the presence of lactose (contained in cheese whey), which contributes to crumb softness for a longer time by acting as a fat emulsifier and maintaining humidity at stable levels due to the water-binding capacity of lactose crystals [4]. Furthermore, hardness was increased (*p* < 0.05) with storage time due to starch retrogradation.

The hardness of control bread, prepared from F70/WWF, ranged from 1.64 to 13.05 N, while that of the respective experimental bread ranged from 0.85 to 5.20 N. The hardness of control bread, made from F70/FM, ranged from 0.56 to 3.23 N, while that of the respective experimental bread ranged from 1.00 to 6.08 N. Flour type M is prepared from hard wheat containing more gluten compared to F70 and WWF, which come from soft wheat. It is possible that the particular behavior (with regard to hardness) of the bread prepared from F70/FM may be related to complexes formed between gluten and lactose. However, this assumption should be further investigated. Finally, the hardness of control bread made from flour F70 ranged from 1.12 to 7.49 N, while that of the respective experimental bread ranged from 0.81 to 6.25 N.

No statistically significant differences (*p* > 0.05) were recorded in values of cohesiveness, springiness, and adhesiveness in experimental breads with respect to control breads (data not shown). The same holds for storage time. These findings contradict those of Kadharmestan et al. [25] and Divya and Rao [12], which reported an increase in hardness of bread by incorporating whey concentrate in bread preparation. Similar results were reported by El-Batawy et al. [15], who substituted water with 50% and 100% cheese whey for the preparation of Egyptian Baladi bread. The hardness of the control bread ranged from 1.188 to 6.973 N, while that of 50% whey-substituted bread ranged from 1.469 to 3.717 N and of 100% whey-substituted bread ranged from 1.274 to 6.029 N during 3 days storage time. Bread cohesiveness was slightly improved only in the 100% whey-substituted bread, the springiness decreased by the replacement of water, without remarkable changes during storage, while adhesiveness was not substantially affected.

### 2.3. Sensory Evaluation

Sensory evaluation (Table 4) showed that experimental breads were superior compared to control breads in color/appearance, taste, texture and odor. The most sensitive sensory parameter for all bread samples proved to be appearance (mold growth). Bread samples were rejected when visible mold growth was recorded on day 4 for control breads and on day 5 for experimental breads.

The results for taste evaluation of breads show that both control and experimental breads were acceptable up to day 4 of storage with the experimental breads recording consistently higher scores compared to controls. The same holds for texture and odor scores. Of the three experimental breads prepared, the F70/FM was judged as the most acceptable scoring 7.8, 7.7, 7.5, and 7.4 for color/appearance, taste, texture, and odor, respectively. Based on the sensory evaluation, the shelf life of the experimental breads was 4–5 days vs. 3–4 days for control breads stored at 24 °C.

The sensory data of the present study are in very good agreement with those of Divya and Rao [12] considering the difference in bread storage temperature (30 vs. 24 °C). According to Latou et al. [11], among odor, taste, and texture, the last proved to be the most sensitive attribute for the evaluation of wheat bread shelf life. Based primarily on texture scores but also on visible signs of mold growth and a lower acceptability score of 5, the above authors reported a shelf life of 4–5 days for control bread (containing no preservatives) stored at 24 °C. This finding is in good agreement with the results of the present work considering the difference in bread storage temperature (20 vs. 24 °C). Our results are also in agreement with those of El-Batawy et al. [15], who reported that the substitution of water with 100% cheese whey in Baladi bread formula enhanced its sensory properties (appearance, taste, and odor).

## 3. Materials and Methods

### 3.1. Breadmaking and Storage

Three different flour compositions were used, corresponding to the most common types of bread on the market: (i) wheat flour type 70%/whole wheat flour (1:1) (F70/WWF), (ii) wheat flour type 70%/wheat flour type M (1:1) (F70/FM) and (iii) straight wheat flour type 70% (F70). The composition of flours used was as follows: (1) F70: moisture 12.5%, protein 12%, carbohydrates 74.3%, fat 0.7%, ash 0.5%, (2) FM: moisture 12.5%, protein 14%, carbohydrates 71.9%, fat 0.8%, ash 0.8%, and (3) WWF: moisture 13.5%, protein 11.5%, carbohydrates 71.2%, fat 2%, ash 1.8%. Conventional wheat bread of the same flour composition prepared using water was taken as the controls. A home-type bread maker was used for the preparation of all types of bread. The mixing time was 25 min, and the baking time was 60 min at 200 °C.

Bread was prepared using 400 g wheat flour, 15 g sugar, 7.5 g salt, 8 g commercial dry yeast, 30 mL olive oil, and 240 mL (for F70/WWF and F70 breads)/260 mL (for F70/FM bread) water or untreated cheese whey. Cheese whey was supplied by DODONI DAIRY Co. Ioannina, Greece. The whey used was the by-product of cheesemaking on that particular day and transferred to the laboratory in a portable ice box within 20 min. The whey composition was as follows: moisture 93.5%, dry matter 6.0%, lactose 4.5%, total protein 0.8%, whey protein 0.65%, ash 0.55% on an as-is basis. The total amount of whey used was (240 + 240 + 260) mL for the preparation of 3 loaves of bread per replicate × 2 replicates = 740 × 2 = 1480 mL. 

Experimental and control breads were stored in low-density polyethylene plastic bags in the laboratory at 24 ± 1 °C for up to 6 days. Sampling was carried out on days 0, 2, 4, and 6 (and 1 h after preparation, time required for bread to cool down). The experiment was terminated when samples were no longer sensorily acceptable in any way (objectionable appearance: mold growth, texture, flavor).

### 3.2. Microbiological Analysis

Bread samples (25 g) were transferred aseptically into individual stomacher bags (Seward Medical, Worthing, UK), containing 225 mL of sterile Peptone Water solution (0.1%) and homogenized in a stomacher (Lab Blender 400, Seward Medical, Worthing, UK) for 60 s. For each sample, appropriate serial decimal dilutions were prepared in Peptone Water solution (0.1%). The amount of 0.1 mL of these serial dilutions of bread homogenates was spread on the surface of dry media. The following groups of microflora were determined according to official protocols [26]: Total Viable Counts (TVC), yeasts/molds and *Bacillus cereus*. TVC were determined using Plate Count Agar (LABM, Heywood, UK) after incubation at 30 °C for 3 days. Yeasts and molds were enumerated using Rose Bengal Chloramphenicol Agar (LABM, Heywood, UK) after incubation at 25 °C for 3 and 5 days respectively in the dark. Finally, *Bacillus cereus* was enumerated using mannitol–egg yolk–polymyxin (MYP) agar (LABM, Heywood, UK) after incubation at 30 °C for 24 h. All plates were examined visually for typical colony types and morphological characteristics associated with each growth medium. In addition, the selectivity of each medium was checked routinely by Gram staining and microscopic examination of smears prepared from randomly selected colonies from all of the media.

### 3.3. Physicochemical Analysis

#### 3.3.1. Determination of pH and Total Titratable Acidity (TTA)

pH was determined by immersing the electrode of an electronic pH-meter Delta OHM HD 3456.2 (Caselle di Selvazzano, Italy) in a suspension consisting of 10 g of each homogenized bread (crust and crumb were homogenized in a home type blender) and 90 mL of distilled water. Moreover, the pH of untreated cheese whey was determined by immersing the pH-meter electrode in 20 mL of untreated cheese whey.

The TTA of each bread sample was determined according to the method of Sanz-Penella et al. [27] and was expressed as mL of NaOH 0.1 N, required for the titration of 10 g of bread (crust and crumb) in the presence of phenolphthalein as indicator. The acidity of the untreated cheese whey was determined according to the official AOAC method [28] and was expressed as % *w*/*v* lactic acid.

#### 3.3.2. Determination of Protein and Lactose Content

The protein content of bread samples and untreated cheese whey was determined according to the Kjeldahl method [29]. Protein content was calculated according to the following formulas:

1. Control breads: % protein = % N × 5.7
2. Untreated cheese whey: % protein = % N × 6.38
3. Experimental breads, F70/WWF and F70: % protein = % N × 5.95
  $\left(\frac{400}{647.2}\times 5.7+\frac{247.2}{647.2}\times 6.38=5.95\right).$
4. Experimental breads, F70/FM: % protein = % N × 5.97
  $\left(\frac{400}{667.8}\times 5.7+\frac{267.8}{667.8}\times 6.38=5.97\right).$

For the conversion of whey milliliters to grams, multiply 240 mL × 1.03 g_whey_/mL= 247.2 g. The factors for the three experimental breads derive from the following ratios: g flour/(g flour + g untreated cheese whey) plus g untreated cheese whey/(g flour + g untreated cheese whey) used for breadmaking i.e., for experimental breads F70/WWF and F70 (400 g of flour, 247.2 g of untreated cheese whey, 647.2 g of flour plus g of untreated cheese whey) and experimental bread F70/FM (400 g of flour, 267.8 g of untreated cheese whey, 667.8 g of flour plus g of untreated cheese whey). The lactose content in the experimental breads and in untreated cheese whey was determined by the chloramine-T method [30]. In brief, a deproteinized sample of the sample is mixed with chloramine-T and KI, and a series of reactions generate KIO within the sample, which reacts with lactose. Acidification of the sample liberates I_2_ from the unreacted KIO and allows its estimation by titration with Na_2_S_2_O_3_ using starch as an indicator.

#### 3.3.3. Semi-Quantitative Determination of Volatile Compounds 

Volatiles were determined on day 0 and 4 (end of shelf life) of storage according to the method of Latou et al. [11] after appropriate modification. First, 1 g of homogenized bread (crust and crumb) and 20 μL of 4-methyl-2-pentanol (324 mg/kg) as an internal standard were placed into a 20 mL glass serum vial and sealed with an aluminum crimp cap provided with a needle-pierceable polytetrafluroethylene/silicone septum. Solid-phase microextraction (SPME) was performed with a 50/30 μm Divinylbenzene/Carboxen/Polydimethyl-siloxane (DVB/CAR/PDMS) fiber mounted to a SPME manual holder assembly (Supelco, Bellefonte, PA, USA). The sample vial was incubated in a 50 °C water bath. After allowing 5 min for the sample to equilibrate at 50 °C, the needle of the SPME device was inserted into the vial through the septum, and the plunger of the SPME apparatus was pushed down to expose the DVB/CAR/PDMS fiber to the vial headspace and sorb the volatiles. After 15 min of exposure time, the fiber was retracted into the needle assembly, removed from the vial, and transferred to the injection port of the GC unit. Desorption of volatiles took place in the injector port (270 °C) of the GC/MS Agilent 7890 GC System (Wilmington, DE, USA) for 10 min in split mode (2:1). The chromatographic column used was Agilent DB-5MS (5% phenylmethylpolysiloxane) with a 0.25 μm film thickness, 60 m × 0.32 μm i.d. The temperature program was 40 °C maintained for 5 min; then, it increased by 10 °C /min to 100 °C, by 5 °C /min to 205 °C, and by 10 °C /min to 260 °C, where it was held for 5 min. The carrier gas was helium, with a column flow of 1.5 mL/min. Source temperature: 230 °C, quadrupole temperature: 150 °C; transfer line temperature: 280 °C. Mass spectra were recorded on a GC-MS 5975C inert XL MSD mass spectrometer (Wilmington, DE, USA) in the mass range *m/z*= 29–350. The above conditions were the result of preliminary optimization including sample weight, fiber type, equilibration time, and temperature. The identification of volatiles was achieved by comparison of the MS data with those in the NIST05 library. For the determination of retention indices, a mixture of *n*-alkanes (C5–C7 and C8–C20) dissolved in *n*-hexane was employed. The mixture was supplied by Fluka (Buchs, Switzerland). The calculation was carried out for components eluting between *n*-pentane and *n*-eicosane.

#### 3.3.4. Color Measurement

Color measurement of each bread sample, crumb and crust separately, was performed using a HunterLab colourimeter equipped with an optical sensor D25 L and a HunterLabDP-9000 processor (Reston, VA, USA). The color for each sample was measured in four points on its surface, based on the CIE Lab system recording color parameters L * = lightness, a * = red/green, b * = yellow/blue.

#### 3.3.5. Texture Analysis

Texture analysis was performed using an Instron Universal Testing Machine, model 4411 (Instron Corp., Bucks, UK). Cylindrical discs of bread crumb (2.5 cm in diameter, 2 cm in height) were cut from the center of each bread slice and were subjected to two compressions in quick succession with withdrawal of the compressing force after each compression, simulating the processes of biting and chewing in the mouth. The conditions of the double compression cycle were as follows: crosshead speed 100 mm/min, specimen compression depth 60%, diameter of stainless steel compression plunger: 7.5 cm.

The texture parameters measured were hardness (peak force of the first compression cycle, N), cohesiveness (strength of the internal bonds in the sample, dimensionless), springiness (rate at which a deformed sample returns to its original size and shape, mm), and adhesiveness (work required to overcome the sticky forces between the sample and the probe, J). Data were recorded using the Bluehill software (Version 1.4, Instron, Norwood, MA, USA).

### 3.4. Sensory Evaluation

Sensory evaluation (acceptability test) was carried out by a 51-member untrained panel consisting of faculty members and graduate students of the Food Chemistry Laboratory, Department of Chemistry, University of Ioannina. Panelists were chosen using the following criteria: ages between 22 and 60, non-smokers, without reported cases of food allergies who consume bread daily. 

Sensory data were collected between day 0 and day 6 of storage for all breads. Approximately 20 g of each bread slice (crust and crumb) were placed in small plastic cups, coded appropriately, and presented to the panelists. The panelists were asked to score color, texture, taste, and odor on a 9-point hedonic scale, where 9 = extremely like and 1 = extremely dislike. A score of 5 was taken as the lower limit of acceptability for each sensory attribute. Bread samples were considered unacceptable upon the first visible sign of mold growth or when receiving a score of less than 5 in any of the attributes evaluated. 

### 3.5. Statistical Analysis

All experiments were replicated twice on different occasions with two determinations carried out per replicate for all analytical parameters determined (*n* = 2 × 2 = 4). Data were subjected to analysis of variance (two-way ANOVA) using the software SPSS 23.0. Pearson’s coefficient was used in order to determine the degree of relationship between two variables. Results were reported as mean value ± standard deviation (S.D.). Significance was defined at *p* < 0.05.

## 4. Conclusions

The results of the present study showed that the incorporation of untreated whey to bread improved its sensory characteristics (more intense flavor), nutritional value (higher protein content), and increased its mold-free shelf life compared to control bread (4–5 days for whey-enriched bread vs. 3–4 days for control bread). Furthermore, the proposed use of whey in bread preparation contributes decisively to the environmentally friendly management of whey responsible for an enormous amount of pollution due to its high BOD and COD content.

## Figures and Tables

**Table 1:** Effect of water substitution by untreated cheese whey and storage time on volatile compounds (μg/kg) of breads, 1: F70/WWF, 2: F70/FM, 3: F70, stored at ambient temperature.

Volatile Compounds of Bread 1	Experimental Bread		Control Bread		KI_Ex_	KI_Li_
	Day 0	Day 4	Day 0	Day 4		
Ethanol	3378.9 ± 223.9	1442.8 ± 321.1	5275.9 ± 394.9	908.8 ± 146.9	<500	<500
2-Methyl-1-propanol	143.2 ± 3.3	n.d.	166.6 ± 12.9	n.d.	625	625
3-Methyl-1-butanol	417.9 ± 5.9	261.1 ± 1.8	619.3 ± 51.8	156.7 ± 23.9	735	731
2-Methyl-1-butanol	191.3 ± 3.7	126.1 ± 10.3	265.2 ± 9.7	n.d.	739	735
2,3-Butanediol	82.9 ± 2.3	69.6 ± 13.4	110.5 ± 50.3	144.2 ± 57.1	779	779
1-Pentanol	21.7 ± 0.2	n.d.	n.d.	n.d.	766	766
1-Hexanol	n.d.	141.1 ± 1.6	122.7 ± 5.8	n.d.	866	862
Phenylethanol	n.d.	n.d.	34.7 ± 0.4	47.9 ± 17.0	1128	1127
2-Furanmethanol	n.d.	14.4 ± 5.9	n.d.	n.d.	852	853
2-Methyl-1-propanal	46.7 ± 3.5	n.d.	n.d.	n.d.	554	553
3-Methyl-1-butanal	115.6 ± 1.2	n.d.	94.2 ± 5.0	n.d.	658	650
Hexanal	295.6 ± 24.0	192.2 ± 22.7	377.7 ± 49.7	195.4 ± 37.8	801	798
Heptanal	133.5 ± 5.0	n.d.	162.1 ± 3.3	71.8 ± 11.2	903	899
Octanal	44.6 ± 4.3	28.9 ± 1.8	n.d.	33.8 ± 0.9	1005	1004
Nonanal	126.5 ± 16.8	164.2 ± 4.6	128.9 ± 0.8	154.7 ± 25.7	1107	1105
Decanal	12.8 ± 6.3	n.d.	n.d.	9.5 ± 0.1	1210	1205
Benzaldehyde	18.4 ± 1.8	18.3 ± 2.5	19.6 ± 2.3	15.2 ± 0.8	977	970
Phenylacetaldehyde	14.5 ± 1.8	n.d.	11.1 ± 1.3	n.d.	1068	1055
Acetoin	447.4 ± 5.9	402.1 ± 7.1	163.9 ± 36.5	152.4 ± 34.2	710	721
6-Methyl-5-hepten-2-one	16.8 ± 0.3	n.d.	n.d.	n.d.	985	985
Methyl butanoate	79.9 ± 6.5	94.0 ± 7.6	103.9 ± 0.4	97.1 ± 9.1	722	735
Ethyl butanoate	57.9 ± 0.9	72.5 ± 1.4	83.2 ± 0.8	74.9 ± 14.1	798	799
Methyl hexanoate	37.5 ± 3.4	40.3 ± 2.3	53.9 ± 12.6	48.3 ± 8.3	921	934
Ethyl octanoate	47.2 ± 6.1	57.2 ± 0.5	81.8 ± 4.4	60.4 ± 6.4	1193	1195
Hexane	3008.2 ± 108.9	3082.5 ± 547.8	3310.8 ± 45.9	2674.5 ± 1.1	595	600
Heptane	n.d.	34.3 ± 6.2	n.d.	n.d.	699	700
Nonane	n.d.	13.3 ± 4.1	n.d.	n.d.	900	900
Decane	47.3 ± 5.1	42.0 ± 7.2	n.d.	13.7 ± 2.4	1000	1000
Tridecane	n.d.	8.0 ± 0.6	11.7 ± 0.6	n.d.	1300	1300
2-Pentyl-furan	75.5 ± 1.1	n.d.	n.d.	n.d.	994	994
α-Pinene	12.9 ± 1.4	9.9 ± 0.0	11.5 ± 2.6	7.6 ± 0.2	947	943
Limonene	468.0 ± 133.1	369.7 ± 43.1	437.4 ± 80.9	326.2 ± 63.6	1042	1039
Farnasene	11.6 ± 2.8	12.8±3.8	15.6 ± 0.3	11.6 ± 2.5	1510	1496
Ocimene	47.4 ± 0.6	25.2 ± 5.2	26.8 ± 2.4	12.8 ± 2.3	1048	1041
*p*-Cymene	56.9 ± 10.1	50.1 ± 4.1	52.1 ± 9.5	43.4 ± 8.6	1037	1034
**Volatile Compounds of Bread 2**	**Experimental Bread**		**Control Bread**		**KI_Ex_**	**KI_Li_**
	**Day 0**	**Day 4**	**Day 0**	**Day 4**		
Ethanol	3495.8 ± 377.4	381.3 ± 34.7	1972.9 ± 329.4	n.d.	<500	<500
2-Methyl-1-propanol	156.8 ± 58.9	n.d.	110.2 ± 26.5	n.d.	625	625
3-Methyl-1-butanol	482.7 ± 75.3	42.1 ± 8.9	283.0 ± 51.9	n.d.	735	731
2-Methyl-1-butanol	246 ± 56.8	n.d.	154.3 ± 27.4	n.d.	739	735
2,3-Butanediol	144.5 ± 40.9	115.9 ± 24.5	108.5 ± 7.0	97.3 ± 9.7	779	779
1-Hexanol	129.8 ± 29.8	81.1 ± 15.2	56.2 ± 3.5	47.1 ± 5.3	866	862
1-Octen-3-ol	n.d.	n.d.	n.d.	105.2 ± 3.7	979	978
Phenylethanol	7.4 ± 2.3	11.6 ± 0.6	17.1 ± 4.2	21.6 ± 7.1	1128	1127
2-Furanmethanol	n.d.	n.d.	28.7 ± 6.4	n.d.	852	853
2-Methyl-1-propanal	n.d.	n.d.	63.8 ± 6.5	n.d.	554	553
2-Methyl-1-butanal	26.0 ± 8.9	n.d.	67.1 ± 11.8	n.d.	667	660
3-Methyl-1-butanal	69.7 ± 11.8	n.d.	170.6 ± 16.5	n.d.	658	650
Hexanal	214.4 ± 26.8	57.1 ± 14.4	203.4 ± 0.2	42.5 ± 7.9	801	798
Heptanal	n.d.	n.d.	n.d.	41.3 ± 4.0	903	899
Octanal	45.3 ± 3.7	16.1 ± 5.1	n.d.	n.d.	1005	1004
Nonanal	162.4 ± 12.6	87.3 ± 23.3	68.1 ± 14.4	30.8 ± 9.9	1107	1105
Benzaldehyde	22.3 ± 2.4	n.d.	13.9 ± 2.2	n.d.	977	970
Phenylacetaldehyde	20.4 ± 1.2	n.d.	9.9 ± 0.9	n.d.	1068	1055
Furfural	n.d.	n.d.	40.3 ± 2.3	n.d.	836	830
Acetoin	530.1 ± 83.0	282.6 ± 85.6	284.9 ± 2.5	148.2 ± 27.9	710	721
6-Methyl-5-hepten-2-one	n.d.	12.5 ± 3.0	n.d.	n.d.	985	985
Methyl butanoate	120.0 ± 20.5	83.3 ± 15.8	90.5 ± 6.3	104.5 ± 26.8	722	735
Ethyl butanoate	73.5 ± 15.6	50.2 ± 17.0	55.6 ± 7.3	57.6 ± 13.0	798	799
Methyl hexanoate	43.7 ± 3.6	77.7 ± 22.9	28.6 ± 5.1	47.3 ± 0.2	921	934
Ethyl hexanoate	n.d.	11.8 ± 4.1	n.d.	13.0 ± 1.9	995	996
Methyl octanoate	n.d.	25.6 ± 11.5	n.d.	22.9 ± 5.0	1121	1129
Ethyl octanoate	47.8 ± 1.8	35.7 ± 5.4	n.d.	24.5 ± 3.8	1193	1195
Methyl decanoate	n.d.	15.7 ± 9.6	n.d.	n.d.	1322	1328
Methylcyclopentane	n.d.	n.d.	n.d.	15.1 ± 1.2	629	635
Hexane	3266.5 ± 93.3	2165.8 ± 354.8	2667.9 ± 170.3	2866.8 ± 277.1	595	600
Heptane	n.d.	23.8 ± 4.1	n.d.	26.9 ± 6.1	699	700
Nonane	11.9 ± 0.6	11.3 ± 2.3	n.d.	n.d.	900	900
Decane	50.6 ± 9.8	41.6 ± 0.5	10.9 ± 2.6	13.4 ± 1.3	1000	1000
1-Decene	n.d.	17.4 ± 1.8	n.d.	n.d.	992	989
2-Pentyl-furan	63.9 ± 5.2	n.d.	32.8 ± 3.0	n.d.	994	994
1,2,3-Trimethylbenzene	n.d.	n.d.	n.d.	10.1 ± 0.3	1007	1002
α-Pinene	8.3 ± 1.8	5.1 ± 0.9	7.2 ± 0.4	6.7 ± 0.1	947	943
Limonene	239.6 ± 6.7	83.7 ± 14.7	190.1 ± 2.4	105.6 ± 14.9	1042	1039
Farnasene	13.5 ± 1.8	10.0 ± 3.8	6.3 ± 0.3	10.6 ± 0.9	1510	1496
Ocimene	36.3 ± 9.2	10.5 ± 0.4	16.9 ± 0.6	n.d.	1048	1041
*p*-Cymene	39.2 ± 0.9	32.0 ± 0.8	28.8 ± 1.8	28.4 ± 3.3	1037	1034
**Volatile Compounds of Bread 3**	**Experimental Bread**		**Control Bread**		**KI_Ex_**	**KI_Li_**
	**Day 0**	**Day 4**	**Day 0**	**Day 4**		
Ethanol	2620.7 ± 163.9	542.2 ± 24.5	3162.7 ± 404.0	259.9 ± 9.4	<500	<500
2-Methyl-1-propanol	85.8 ± 10.9	n.d.	137.9 ± 25.0	n.d.	625	625
3-Methyl-1-butanol	253.8 ± 33.9	97.1 ± 3.5	502.6 ± 27.0	42.9 ± 3.5	735	731
2-Methyl-1-butanol	131.0 ± 16.9	51.5 ± 1.1	224.6 ± 16.1	n.d.	739	735
2,3-Butanediol	228.9 ± 48.0	105.4 ± 11.1	116.7 ± 33.1	159.1 ± 18.7	779	779
1-Hexanol	45.7 ± 8.7	38.8 ± 2.9	41.4 ± 1.1	33.7 ± 3.2	866	862
Phenylethanol	n.d.	n.d.	40.3 ± 13.2	57.7 ± 7.4	1128	1127
2-Furanmethanol	66.9 ± 28.1	n.d.	n.d.	n.d.	852	853
2-Methyl-1-propanal	62.1 ± 10.2	n.d.	n.d.	n.d.	554	553
2-Methyl-1-butanal	74.6 ± 16.5	n.d.	45.6 ± 11.0	n.d.	667	660
3-Methyl-1-butanal	129.5 ± 34.3	n.d.	93.6 ± 13.6	5.8 ± 0.4	658	650
Hexanal	171.6 ± 31.6	n.d.	122.3 ± 15.6	n.d.	801	798
Heptanal	83.9 ± 11.0	21.5 ± 2.5	59.1 ± 9.6	14.9 ± 3.3	903	899
Octanal	46.8 ± 16.3	n.d.	n.d.	n.d.	1005	1004
Nonanal	91.4 ± 19.7	n.d.	55.9 ± 1.6	11.3 ± 2.4	1107	1105
Benzaldehyde	14.0 ± 4.4	n.d.	19.4 ± 0.8	n.d	977	970
Phenylacetaldehyde	n.d.	n.d.	12.7 ± 1.7	n.d.	1068	1055
Furfural	97.5 ± 13.8	n.d.	n.d.	n.d.	836	830
Acetoin	419.1 ± 56.9	200.2 ± 17.8	111.2 ± 0.4	26.2 ± 4.2	710	721
3-Methyl-2-butanone	106.7 ± 17.2	n.d.	n.d.	n.d.	585	590
Methyl butanoate	116.9 ± 0.1	67.2 ± 0.6	78.5 ± 5.7	57.9 ± 7.6	722	735
Ethyl butanoate	65.7 ± 3.5	35.5 ± 2.1	45.4 ± 4.9	32.3 ± 4.7	798	799
Methyl hexanoate	46.0 ± 2.1	32.2 ± 4.3	44.2 ± 10.3	14.6 ± 1.3	921	934
Ethyl octanoate	39.3 ± 5.7	29.2 ± 2.3	n.d.	23.5 ± 1.3	1193	1195
Hexane	2698.7 ± 58.6	1876.5 ± 59.4	2110.1 ± 367.9	1829.9 ± 149.8	595	600
Heptane	n.d.	15.4 ± 0.1	n.d.	12.6 ± 1.1	699	700
Nonane	18.0 ± 1.9	7.3 ± 2.9	n.d.	n.d.	900	900
Decane	52.8 ± 2.8	27.9 ± 4.7	18.8 ± 2.6	21.8 ± 0.2	1000	1000
Undecane	10.6 ± 2.8	n.d.	n.d.	n.d.	1100	1100
1-Decene	29.8 ± 10.8	n.d.	n.d.	n.d.	992	989
2-Acetyl-furan	18.4 ± 6.6	n.d.	n.d.	n.d.	915	907
2-Pentyl-furan	n.d.	n.d.	34.5 ± 0.9	n.d.	994	994
2-Ethyl-5-methyl-pyrazine	n.d.	n.d.	13.6 ± 5.8	n.d.	1010	998
2,5-Dimethyl-pyrazine	n.d.	n.d.	50.8 ± 20.8	n.d.	918	908
α-Pinene	8.4 ± 0.5	4.7 ± 0.9	6.2 ± 0.3	3.9 ± 0.4	947	943
Limonene	75.5 ± 10.01	41.6 ± 0.3	81.3 ± 12.2	37.2 ± 3.6	1042	1039
Farnasene	11.0 ± 1.6	5.9 ± 1.5	7.9 ± 2.8	5.4 ± 0.3	1510	1496
Ocimene	21.8 ± 1.6	n.d.	18.3 ± 3.7	4.9 ± 1.1	1048	1041
*p*-Cymene	27.1 ± 2.4	29.4 ± 1.3	27.1 ± 1.2	26.9 ± 4.2	1037	1034

n.d. = not detected. Values are the mean of four determinations (*n* = 4). KI_Ex_ = Kovac Index Experimentally determined data, KI_Li_ = Kovac Index Literature data Nist 05.

**Table 2:** Effect of water substitution with untreated cheese whey and storage time on color parameters of breads, 1: F70/WWF, 2: F70/FM, 3: F70, stored at ambient temperature.

Days of Storage	Control 1 (Crumb)	Experimental 1 (Crumb)	Control 2 (Crumb)	Experimental 2 (Crumb)	Control 3 (Crumb)	Experimental 3 (Crumb)
	L *					
0	58.05 ± 0.06 ^aB^	63.40 ± 0.11 ^bB^	73.42 ± 0.16 ^bA^	69.81 ± 0.08 ^aA^	75.97 ± 0.11 ^aD^	76.51 ± 0.18 ^bB^
2	59.03 ± 0.20 ^aC^	60.65 ± 1.96 ^aA^	73.85 ± 0.14 ^aB^	80.58 ± 0.22 ^bD^	75.57 ± 0.05 ^aB^	76.20 ± 0.05 ^bA^
4	60.13 ± 0.21 ^aD^	59.39 ± 1.80 ^aA^	76.90 ± 0.07 ^aC^	77.25 ± 0.04 ^bC^	74.81 ± 0.04 ^aA^	77.96 ± 0.03 ^bC^
6	55.87 ± 0.28 ^aA^	63.31 ± 0.39 ^bB^	76.89 ± 0.12 ^bC^	73.40 ± 0.04 ^aB^	75.73 ± 0.06 ^aC^	76.17 ± 0.05 ^bA^
	a *					
0	4.91 ± 0.27 ^aA^	5.64 ± 0.50 ^aB^	−0.88 ± 0.20 ^bA^	−1.50 ± 0.09 ^aB^	−7.02 ± 0.32 ^aA^	−5.57 ± 0.26 ^bA^
2	5.54 ± 0.08 ^bB^	4.51 ± 0.36 ^aB^	0.69 ± 0.12 ^aC^	−1.33 ± 0.14 ^bB^	−4.06 ± 0.42 ^aC^	0.72 ± 0.17 ^bD^
4	5.11 ± 0.19 ^aA^	5.09 ± 0.34 ^aB^	−0.59 ± 0.13 ^aA^	−2.00 ± 0.09 ^bA^	−5.55 ± 0.62 ^aB^	−2.67 ± 0.17 ^bB^
6	5.95 ± 0.21 ^bC^	2.61 ± 0.91 ^aA^	−0.44 ± 0.32 ^aA^	−0.47 ± 0.21 ^aC^	−4.59 ± 0.35 ^aC^	−2.24 ± 0.23 ^bC^
	b *					
0	25.09 ± 0.14 ^bC^	23.85 ± 0.23 ^aA^	28.66 ± 0.14 ^aC^	29.52 ± 0.10 ^bC^	28.82 ± 0.09 ^aD^	30.82 ± 0.17 ^bC^
2	23.05 ± 0.10 ^aA^	24.56 ± 0.14 ^bB^	26.04 ± 0.09 ^aB^	28.72 ± 0.18 ^bB^	24.58 ± 0.16 ^aA^	25.91 ± 0.05 ^bB^
4	22.84 ± 0.13 ^aA^	24.24 ± 0.22 ^bAB^	28.80 ± 0.23 ^aC^	30.58 ± 0.07 ^bD^	27.42 ± 0.17 ^bC^	24.25 ± 0.27 ^aA^
6	23.63 ± 0.11 ^aB^	23.53 ± 0.45 ^aA^	25.31 ± 0.10 ^aA^	27.35 ± 0.16 ^bA^	24.90 ± 0.11 ^bB^	24.00 ± 0.32 ^aA^
	**Control 1** **(Crust)**	**Experimental 1** **(Crust)**	**Control 2** **(Crust)**	**Experimental 2** **(Crust)**	**Control 3** **(Crust)**	**Experimental 3** **(Crust)**
	L *					
0	55.00 ± 0.26 ^bC^	44.90 ± 0.29 ^aC^	54.93 ± 0.08 ^bC^	42.90 ± 0.03 ^aA^	67.56 ± 0.14 ^bC^	54.59 ± 0.34 ^aC^
2	53.16 ± 0.09 ^bB^	42.25 ± 0.12 ^aB^	54.47 ± 0.12 ^bΒ^	48.16 ± 0.26 ^aC^	65.49 ± 0.20 ^bB^	52.41 ± 0.14 ^aA^
4	52.00 ± 0.22 ^bA^	41.24 ± 0.12 ^aA^	54.11 ± 0.21 ^bA^	44.49 ± 0.73 ^aB^	64.61 ± 0.60 ^bA^	53.53 ± 0.16 ^aB^
6	53.23 ± 0.20 ^bB^	44.45 ± 0.29 ^aC^	54.24 ± 0.08 ^bA^	42.94 ± 0.12 ^aA^	64.79 ± 0.33 ^bA^	52.57 ± 0.26 ^aA^
	a *					
0	12.48 ± 0.18 ^aB^	14.63 ± 0.25 ^bC^	12.08 ± 0.48 ^aA^	11.76 ± 0.31 ^aA^	1.84 ± 0.37 ^aA^	6.73 ± 0.25 ^bA^
2	12.27 ± 0.37 ^aAB^	12.86 ± 0.58 ^aB^	14.23 ± 0.22 ^bB^	13.04 ± 0.25 ^aB^	4.41 ± 0.31 ^aC^	14.88 ± 0.62 ^bC^
4	13.00 ± 0.63 ^aB^	13.26 ± 0.33 ^aB^	12.72 ± 0.41 ^aAB^	11.42 ± 0.46 ^aA^	3.26 ± 0.55 ^aΒ^	11.52 ± 1.22 ^bB^
6	11.84 ± 0.43 ^bA^	9.56 ± 0.62 ^aA^	13.67 ± 0.38 ^aB^	13.24 ± 0.15 ^aB^	4.83 ± 0.46 ^aC^	14.30 ± 1.07 ^bC^
	b *					
0	30.92 ± 0.49 ^bC^	25.04 ± 0.18 ^aA^	35.20 ± 0.10 ^bC^	33.14 ± 0.16 ^aC^	39.68 ± 0.14 ^bC^	46.58 ± 0.28 ^aD^
2	26.76 ± 0.26 ^aA^	26.38 ± 0.61 ^aB^	29.77 ± 0.14 ^aA^	32.42 ± 0.20 ^bB^	31.24 ± 2.13 ^aA^	33.92 ± 0.72 ^aC^
4	28.71 ± 0.26 ^bB^	26.96 ± 0.14 ^aB^	40.76 ± 0.44 ^aD^	45.32 ± 0.70 ^bD^	35.15 ± 0.36 ^bB^	30.69 ± 1.43 ^aΒ^
6	30.59 ± 0.14 ^bC^	29.46 ± 0.55 ^aC^	32.18 ± 0.57 ^bB^	30.40 ± 0.79 ^bA^	32.79 ± 0.34 ^bA^	27.83 ± 1.29 ^aA^

Values are the mean of four measurements (*n* = 4). Means with different lower case superscripts in the same row are statistically different (*p* < 0.05) between control and experimental bread. Means with different capital superscripts in the same column are statistically different (*p* < 0.05) during storage. Color parameters: L = lightness, a = redness/greenness, b = yellowness/blueness.

**Table 3:** Effect of water substitution with untreated cheese whey and storage time on bread stored at ambient temperature.

Flour Type 70%/Whole Wheat Flour (1:1)		
Days of Storage	Hardness (N)	
	Control Bread	Experimental Bread
0	1.64 ± 0.48 ^bA^	0.85 ± 0.13 ^aA^
2	6.11 ± 0.72 ^bB^	2.86 ± 0.34 ^aB^
4	6.63 ± 1.29 ^bB^	4.12 ± 0.82 ^aC^
6	13.05 ± 3.49 ^bC^	5.20 ± 0.80 ^aC^
**Flour Type 70%/Flour Type M (1:1)**		
**Days of Storage**	**Hardness (N)**	
	**Control Bread**	**Experimental Bread**
0	0.56 ± 0.12 ^aA^	1.00 ± 0.50 ^aA^
2	2.20 ± 0.65 ^aB^	1.77 ± 0.49 ^aA^
4	2.63 ± 0.82 ^aB^	4.11 ± 1.39 ^aB^
6	3.23 ± 0.77 ^aB^	6.08 ± 0.67 ^bB^
**Flour Type 70%**		
**Days of Storage**	**Hardness (N)**	
	**Control Bread**	**Experimental Bread**
0	1.12 ± 0.20 ^aA^	0.81 ± 0.11 ^aA^
2	3.20 ± 0.44 ^aB^	2.52 ± 0.35 ^aB^
4	4.78 ± 0.83 ^aC^	3.25 ± 1.71 ^aBC^
6	7.49 ± 2.11 ^aC^	6.25 ± 2.84 ^aC^

Values are the mean of ten measurements (*n* = 10). Means with different lower case superscripts in the same row are statistically different (*p* < 0.05) between control and experimental bread. Means with different capital superscripts in the same column are statistically different (*p* < 0.05) during storage.

**Table 4:** Effect of water substitution with untreated cheese whey and storage time on sensory properties of breads, 1: F70/WWF, 2: F70/FM, 3: F70, stored at ambient temperature.

Days of Storage	Control 1	Experimental 1	Control 2	Experimental 2	Control 3	Experimental 3
**Color/Appearance**						
0	8.8 ± 0.4 ^aA^	9.0 ± 0.0 ^aB^	8.6 ± 1.3 ^aA^	9.0 ± 0.9 ^aA^	8.8 ± 0.4 ^aA^	8.7 ± 0.8 ^aA^
2	8.2 ± 0.5 ^aA^	8.8 ± 0.8 ^aAB^	8.0 ± 1.1 ^aA^	8.2 ± 0.8 ^aA^	8.0 ± 0.8 ^aA^	8.2 ± 0.9 ^aA^
4	mold growth	7.0 ± 1.5 ^aA^	mold growth	7.8 ± 0.8 ^aA^	mold growth	7.3 ± 0.8 ^aA^
6	mold growth	mold growth	mold growth	mold growth	mold growth	mold growth
**Τaste**						
0	8.2 ± 0.4 ^aA^	8.5 ± 0.8 ^aB^	7.8 ± 1.3 ^aA^	9.0 ± 0.9 ^aA^	7.8 ± 0.4 ^aB^	8.5 ± 0.8 ^aB^
2	7.2 ± 1.1 ^aA^	7.6 ± 0.9 ^aAB^	6.9 ± 1.2 ^aA^	8.5 ± 0.8 ^aA^	6.6 ± 0.6 ^aA^	7.8 ± 0.8 ^aAB^
4	-	6.0 ± 1.2 ^aA^	-	7.7 ± 1.0 ^aA^	5.7 ± 0.8 ^aA^	6.2 ± 0.9 ^aA^
6	-	-	-	-	-	-
**Τexture**						
0	8.5 ± 0.5 ^aA^	8.8 ± 0.4 ^aB^	7.8 ± 1.5 ^aA^	8.6 ± 1.3 ^aA^	8.2 ± 1.2 ^aA^	8.3 ± 1.2 ^aA^
2	7.4 ± 0.6 ^aA^	7.4 ± 0.9 ^aA^	6.9 ± 1.2 ^aA^	8.0 ± 1.1 ^aA^	7.0 ± 0.0 ^aA^	7.7 ± 0.8 ^aA^
4	-	6.4 ± 1.1 ^aA^	-	7.5 ± 1.4 ^aA^	-	6.2 ± 1.1 ^aA^
6	-	-	-	-	-	-
**Odor**						
0	8.6 ± 0.5 ^aB^	8.8 ± 0.4 ^aB^	8.4 ± 1.2 ^aA^	8.0 ± 1.7 ^aA^	8.2 ± 0.8 ^aA^	8.5 ± 0.8 ^aA^
2	7.2 ± 0.8 ^aA^	7.4 ± 1.5 ^aAB^	6.8 ± 0.8 ^aA^	7.4 ± 1.2 ^aA^	6.4 ± 1.1 ^aA^	8.4 ± 0.9 ^aA^
4	-	5.8 ± 1.5 ^aA^	-	7.4 ± 0.8 ^aA^	-	6.8 ± 1.2 ^aA^
6	-	-	-	-	-	-

Values are the mean of a 51-member panel (*n* = 51). Means with different lower case superscripts in the same row are statistically different (*p* < 0.05) between control and experimental bread. Means with different capital superscripts in the same column are statistically different (*p* < 0.05) during storage.

## Data Availability

The data presented in this study are available on request from the corresponding authors.

## Sample Availability

Samples of all the ingredients used in the present study are available from the authors.
